# Video Analysis and Verification of Direct Head Impacts Recorded by Wearable Sensors in Junior Rugby League Players

**DOI:** 10.1186/s40798-021-00353-3

**Published:** 2021-09-16

**Authors:** Lauchlan Carey, Douglas P. Terry, Andrew S. McIntosh, Peter Stanwell, Grant L. Iverson, Andrew J. Gardner

**Affiliations:** 1 Centre for Stroke and Brain Injury, School of Health Sciences, Faculty of Health, University of Newcastle, Callaghan, New South Wales Australia; 2 Department of Physical Medicine and Rehabilitation, Harvard Medical School, Boston, Massachusetts USA; 3 Spaulding Rehabilitation Hospital, Boston, Massachusetts USA; 4 MassGeneral Hospital for Children™ Sports Concussion Program, Boston, Massachusetts USA; 5 Home Base, A Red Sox Foundation and Massachusetts General Hospital Program, Charlestown, Massachusetts USA; 6 School of Engineering and Australian Collaboration for Research into Injury in Sport and its Prevention, Edith Cowan University, Perth, Australia; 7 Hunter New England Local Health District Sports Concussion Program, Waratah, NSW Australia; 8 Priority Research Centre for Stroke and Brain Injury, School of Medicine and Public Health, Callaghan, NSW Australia; 9 Hunter Medical Research Institute, New Lambtom Height, NSW Australia

**Keywords:** Head impacts, Rugby league, Wearable sensors, Accelerometer, Video review

## Abstract

**Background:**

Rugby league is a high-intensity collision sport that carries a risk of concussion. Youth athletes are considered to be more vulnerable and take longer to recover from concussion than adult athletes.

**Purpose:**

To review head impact events in elite-level junior representative rugby league and to verify and describe characteristics of X-patch^TM^-recorded impacts via video analysis.

**Study Design:**

Observational case series.

**Methods:**

The X-patch^TM^ was used on twenty-one adolescent players (thirteen forwards and eight backs) during a 2017 junior representative rugby league competition. Game-day footage, recorded by a trained videographer from a single camera, was synchronised with X-patch^TM^-recorded timestamped events. Impacts were double verified by video review. Impact rates, playing characteristics, and gameplay situations were described.

**Results:**

The X-patch^TM^-recorded 624 impacts ≥ 20g between game start and finish, of which 564 (90.4%) were verified on video. Upon video review, 413 (73.2%) of all verified impacts ≥ 20g where determined to be direct head impacts. Direct head impacts ≥ 20g occurred at a rate of 5.2 impacts per game hour; 7.6 for forwards and 3.0 for backs (range = 0–18.2). A defender’s arm directly impacting the head of the ball carrier was the most common event, accounting for 21.3% (*n* = 120) of all impacts, and 46.7% of all “hit-up” impacts. There were no medically diagnosed concussions during the competition.

**Conclusion:**

The majority (90.4%) of head impacts ≥ 20g recorded by the X-patch^TM^ sensor were verified by video. Double verification of direct head impacts in addition to cross-verification of sensor-recorded impacts using a secondary source such as synchronised video review can be used to ensure accuracy and validation of data.

## Key Points

- There was a substantial number of false-positive high acceleration impacts recorded that occurred before, during, or after the games. Wearable instrumented technology has limitations as a primary data source and should be used in conjunction with video review.
- The vast majority of high acceleration impacts (≥ 20g) that occurred during game time were verified on video review.
- Careful time synchronisation of impact sensor-recorded events and match video is vital to help cross-validation and to reduce over-estimation of an athlete’s direct head impact exposure.

## Background

Rugby league carries a risk of concussion due to its high intensity and frequency of collisions [1]. Youth athletes may be more vulnerable to sustaining a concussion [2–4] and may also take longer to recover from a concussion than adult athletes [5–8]. Recently, various technology has been introduced to assist in the identification of head impacts and suspected concussions during athlete competitions. For instance, sideline video review [9, 10], and to a lesser extent, impact sensors in helmeted and non-helmeted sports have been introduced to measure kinematic forces to the head [11, 12].

Sideline video review has become increasingly common in professional sports for identifying head impact events and potential concussions. Recently, multiple experts from seven national and international professional sporting codes developed international consensus definitions of video signs of possible concussion, agreeing on six video signs: (i) lying motionless (for > 2 s); (ii) motor incoordination (e.g. losing balance); (iii) impact seizure; (iv) tonic posturing (involuntary sustained contraction of one or more limbs); (v) no protective action/floppy; and (vi) blank/vacant look [9]. The National Rugby League (NRL) has incorporated a Head Injury Assessment (HIA) process that uses sideline video review as a method to identify direct head impacts and potential signs of concussion in players. The identification of a player displaying potential signs of concussion evokes the HIA process, which includes mandatory immediate removal from play and subsequent assessment [13]. During the 2014 season, the incidence of suspected concussions based on the use of this process was 24.0 per 1000 NRL player game hours [13]. In the same season, the incidence of medically diagnosed concussions following the use of this process was 8.9 per 1000 player game hours [14].

Another proposed method for ascertaining whether a possible concussion occurred during gameplay has been measuring the kinematic responses of a player’s head to impact forces through wearable sensor technology. The X2 X-patch^TM^ is an impact sensor designed for non-helmeted athletes that has been used in three previous rugby league studies in junior, women’s, and semi-professional competitions [15–17]. Worn behind the ear, the X-patch^TM^ uses a triaxial gyroscope and accelerometer to calculate linear and angular accelerations experienced by the head during collisions [18]. Previous X-patch^TM^ studies in under 10-year-old rugby league [15] and under 9-year-old rugby union [19] reported on impact magnitudes comparable to studies on young adults. However, given that the X-patch-recorded impacts were not verified on video, the validity of these findings is questionable [20]. Some studies have examined *helmeted* impacts in 15- to 17-year-old athletes (e.g. American Football [21–24], Lacrosse [18]) using wearable sensors, but no studies have examined impacts in similarly aged rugby league players. Given that a direct head impact is more likely to result in a concussion than an indirect head impact [25], the relevance of player characteristics and gameplay situations to the relative risk of sustaining concussion may be an important consideration. The purpose of this study is to (i) determine the rate at which sensor-recorded impacts using the X-patch^TM^ are verified on video review of game footage, (ii) document the number of video verified direct head impacts that are not recorded on the sensors, and (iii) describe and compare playing characteristics and gameplay situations of video-verified direct and indirect impacts over a season of play in a squad of elite-level youth (under 16s) rugby league players.

## Methods

### Participants

A prospective cohort study was performed on a junior male representative rugby league team during the 2017 New South Wales (NSW) Rugby League Harold Matthews Competition. The Harold Matthews competition is an elite-level, state-based season of games for under 16-year-old male rugby league players. It forms one of the first stages of the elite-level pathway. The competition consists of 16 clubs from the NRL and Canterbury (NSW) Cup competitions. The Harold Matthews competition is played over 9 weeks, with the top five teams qualifying for the post-season (i.e. a 3-week finals series). From a squad of 22 adolescent players, 21 (age range: 15–16 years, mean = 15.5 years, SD = 0.5 years) including 13 forwards and 8 backs from one club participated in the study, with one player declining to participate. Written consent was obtained by a legal parent or guardian for each participating player, and verbal assent was obtained by each individual player. A rugby league team consists of 13 players (6 forwards and 7 backs) on the field at any one time with 4 interchange players. On average, data were collected from 13 participants per week (range: 9–15 players per week).

The research protocol was approved by the University of Newcastle Human Research Ethics Committee. The study was also endorsed by the participating club. The methods for data collection were identical to our previous study on a semi-professional men’s rugby league team [17].

### Measures

#### Impact Sensors

A total of 15 X-patch^TM^ sensors (X2 Biosystems) were available and deployed at the beginning of the season. Each sensor contains a low-power, high-g triaxial accelerometer, and gyroscope that measures linear and angular accelerations and decelerations to provide 6 degrees of freedom kinematic head impact data. All players’ sensors were attached to the skin covering the right mastoid process by an experienced member of the research team. Positioning of the sensor is crucial to ensure it is not activated by soft tissue muscles in the neck [26]. Each sensor was uniquely labelled and attached before the warm up using a double-sided adhesive patch. The X-patch^TM^ is triggered when linear acceleration exceeds 10g and records data for 90 ms after the trigger and 10 ms before the trigger equalling one-tenth of a second of data (100 ms) to its on-board memory. Once the sensor is removed, its stored data can be downloaded and analysed using the Injury Management Software (IMS; X2 Biosystems). Each recorded event is “timestamped” and a set of impact measures are recorded, including PLA, peak rotational acceleration (PRA), peak rotational velocity (PRV), and head impact location. In our study, a Head Impact (HI ≥ 20g) was defined as an event recorded by the X-patch^TM^ with a peak linear acceleration (PLA) ≥ 20g. Emphasis was placed on impacts ≥ 20g to avoid confusion with a large number of low-acceleration events, unlikely to result in deleterious neurophysiological change [17, 27]. Brennan et al [28] observed that the mean PLA associated with concussion was 99g. PLA was utilised in this as previous research shows it has greater reliability and less variance than rotational measurements [20, 29]. To remove low-acceleration events commonly associated with normal gameplay (e.g. sharp changing of directions, jumping, running) all video-verified impacts were filtered to only include HI ≥ 20g as suggested in previous studies [17, 27, 30].

Sensors were collected from players after the game. All recorded impacts were reviewed and extracted from IMS, displayed in the form of a Microsoft Excel spreadsheet, and sorted into individual player cross-tabulations. Each sensor was then cleared of all impacts and charged in preparation for the following game.

#### Video Review and Synchronising with Sensors’ Time Stamp

Each game was recorded with a single high-definition camera by a trained videographer. The video closely followed the play, including both the ball carrier and engaged defenders, and therefore captured competition-related collisions. The best possible vantage point was obtained on the midline of the field with close-up shots panning left and right to follow the play. Each game was reviewed from start to finish using QuickTime X (Apple Inc.) by one reviewer (LC). Video was synchronised with the timestamps of each sensor before the verification review was conducted. The first head impact seen on video review was checked against the HI ≥ 20g after the game start time on the sensor’s timestamp, the same synchronisation method used previously on collegiate Lacrosse athletes by Kindschi and colleagues [31]. Subsequent video-recorded impacts were then checked against timestamps at corresponding intervals. To synchronise the timestamp from the X-patch^TM^ with the video footage time, multiple impacts were reviewed on video and aligned to the sensor timestamp. Each potential video verified HI ≥ 20g (VV-HI ≥ 20g) was checked multiple times with both the timestamp and video to establish they were precisely synchronised before conducting the video verification process from start to finish of gameplay.

VV-HI ≥ 20g were also classified by the game event or situation. Each VV-HI ≥ 20g was deemed to be a “Hit-Up” (attacking player carrying the ball), “Tackle” (defending player attempting to stop the ball carrier), or “Off-The-Ball” incident (contact without the ball). Triggered events ≥ 20g that did not correlate with a collision on video review were documented. Similarly, collisions on video review that involved a player with a working sensor attached and did not correspond to a triggered event were documented. For this study a “Direct VV-HI ≥ 20 g” was defined as a clearly observed physical head contact that corresponded with a HI ≥ 20g, whereas an “Indirect VV-HI ≥ 20 g” was a clearly observed body contact, excluding the head, that corresponded with a HI ≥ 20g. VV-HI ≥ 20g were then sorted into a number of sub-categories including the following: (i) direct (impact to head) vs. non-direct, (ii) number of tacklers involved (i.e. 1–4), (iii) point of impact on player with sensor (i.e. head, shoulder, chest, arm, waist and below), (iv) side of impact (i.e. right, left, front, back, top), (v) point of contact from opposition player (i.e. head, shoulder, chest, arm, waist and below), and (vi) wrestling impacts happening after first initial contact from tackle. A second reviewer (AG) then independently reviewed HI ≥ 20g during game time that were *not verified on video*. The process of double verification of these “false-positive” impacts helped clarify the accuracy of each ‘impact’ included in the video verified data. Using the synchronised data set, the timestamps of non-verified HI ≥ 20g were double-checked with the corresponding video time. Video for approximately 20 s before and after the HI ≥ 20g was reviewed with a focus on the relevant player. The results of this review process were then coded into categories (e.g. “HI ≥ 20g not fully visualised in the available footage”, or “HI ≥ 20g fully visualised with no contact identified”). All HI ≥ 20g cases that were not verified as involving either a direct or indirect impact were excluded from the analyses. This double verification process was conducted independently by the two reviewers.

### Statistical Analyses

Descriptive statistics for PLA and PRV of VV-HI ≥ 20g were calculated and included frequencies, percentages, medians, and standard deviations. VV-HI ≥ 20g per player game hours rates were calculated for all players and positions using the number of VV-HI ≥ 20g divided by the number of game hours. The formula for calculating the impact rate is provided below.

$$ \mathrm{Impact}\ \mathrm{Rate}=\sum \mathrm{VV}-\mathrm{HI}\ge 20\mathrm{g} $$

$$ \sum \mathrm{Player}\ \mathrm{Game}\ \mathrm{Hours} $$

Percentages of video verified and non-verified HI ≥ 20g were calculated to determine the validity of the X-patch^TM^ and to remove any “false positives” from the analysed data set. This was calculated as the number of VV-HI ≥ 20g divided by the number of total recorded HI ≥ 20g during gameplay, multiplied by one hundred. The formula for calculating percentage of verified impacts is provided below.

$$ \%\mathrm{Video}\ \mathrm{Verified}\ \mathrm{Impacts}=\sum \mathrm{VV}-\mathrm{HI}\ge 20\mathrm{g}\times 100 $$

$$ \sum \mathrm{HI}\ge 20\mathrm{g} $$

Location accuracy of direct and indirect VV-HI ≥ 20g was analysed and the accuracy percentage was calculated to show the agreement between the VV-HI ≥ 20g impact location (i.e. front, back, side, top) estimated from the sensor data in the IMS and video review. Location accuracy percentages were calculated as the number of times the sensor-based and the video-based location estimates were in agreement divided by the total number of impacts per location on video review, multiplied by one hundred. The formula for location accuracy for VV-HI ≥ 20g is provided below.

$$ \mathrm{Location}\ \mathrm{Accuracy}\kern0.17em \mathrm{for}\ \mathrm{VV}-\mathrm{HI}\ge 20\mathrm{g}\kern0.5em =\kern0.5em \sum \mathrm{location}\ \mathrm{agreement}\kern0.5em \times 100 $$

$$ \sum \mathrm{total}\kern0.34em \mathrm{video}\kern0.34em \mathrm{locations} $$

VV-HI ≥ 20g data were reviewed for playing positions (i.e. forward versus back) and characteristics (i.e. attacking, defending, off-the-ball). An identical approach to our previous video verification study [17] for the analysis of this data was conducted. VV-HI ≥ 20g incidence rates for forwards and backs were compared using an exploratory *t* test. Exploratory Mann-Whitney *U* tests compared impact magnitude (i.e. PLA, PRV) between video-verified/non-verified HI ≥ 20g, direct/indirect VV-HI ≥ 20g, first/second half VV-HI ≥ 20g, and forward/back position VV-HI ≥ 20g because these variables were not normally distributed. All analyses were performed using SPSS 23 (IBM Corp).

## Results

### Game Hours and Sensor Recording

A total of 79.4 player game hours (4762 min) was recorded, with backs accounting for 52.1% (2479 min) and forwards accounting for 47.9% (2283 min) of the hours. Throughout the season, the number of available and working sensors was reduced to eleven due to deteriorating battery life (i.e. the sensor did not recharge), or the sensor was permanently lost during a game. The X-patch^TM^ became detached 16 times throughout the season from eight different players (2 players once, 5 players twice, and 1 player 4 times), for a total of 456 min of lost data due to detached sensors for the season (backs: *n* = 4, total = 148 min, mean = 37 min, median = 33 min, SD = 11.11, range = 29–53; forwards: *n* = 12, total = 308, mean = 25.7 min, median = 28.5 min, SD = 9.94, range = 11–45). In addition, there was 121 min of game time lost due to 3 faulty sensors (all forwards).

### Sensor-Recorded Impacts

**Fig. 1:**
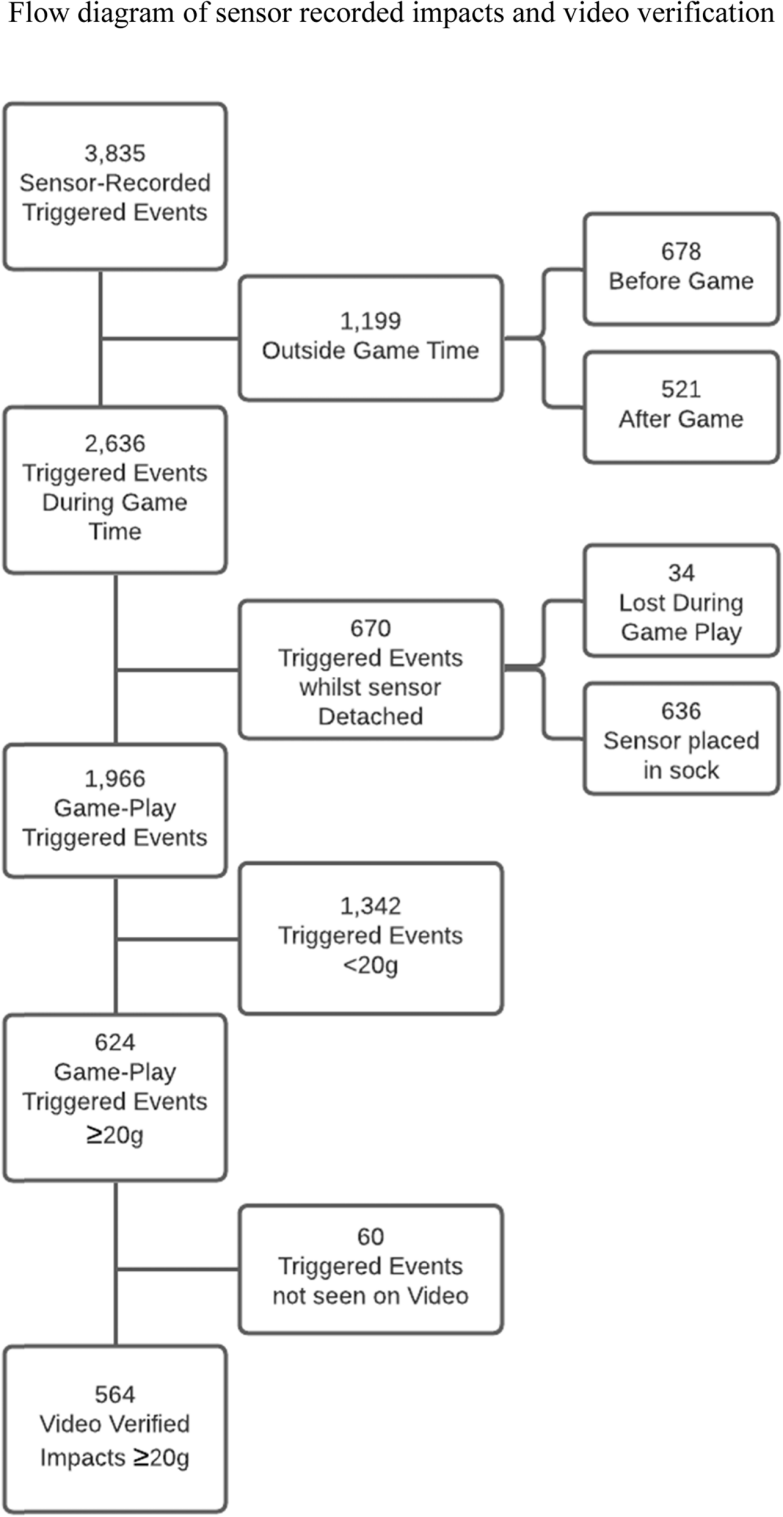
Flow diagram of sensor recorded impacts and video verification

There were 3835 triggered events recorded by the X-patch^TM^ with PLA ≥10g (see Fig. 1). Triggered events that could be interpreted as head impacts that occurred outside of game time (i.e. in warm up, cool-down, during application/removal of sensors) accounted for 1199 impacts (31.3%; 678 before game, 521 after game). On video review, 34 triggered events were removed due to occurring in the process of, or after, the sensor becoming detached in a tackle. A further 636 triggered events were removed due to two players placing the sensor in their sock after it became dislodged leaving a total of 1966 triggered events with PLA ≥ 10g during gameplay. From these triggered events, 1342 were < 20g and therefore excluded which yielded a total of 624 triggered events ≥ 20g during gameplay (HI ≥ 20g).

### Video Verification of Sensor-Recorded Impacts

**Fig. 2:**
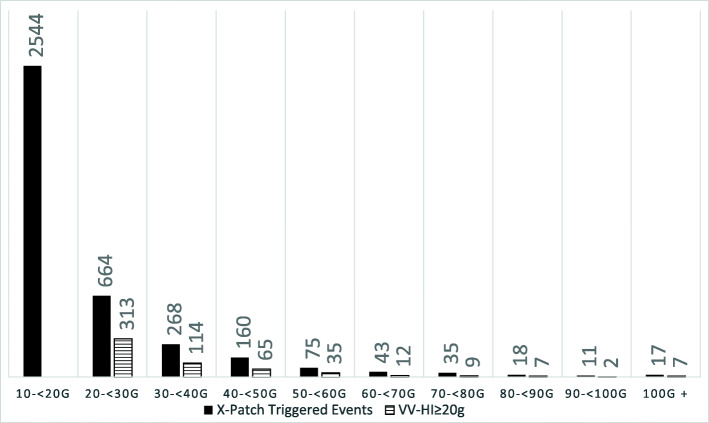
Distribution of all X-patch^TM^ triggered events and VV-HI ≥ 20g. The black bar denotes X-patch^TM^ triggered events. The white bar with stripes denotes VV-HI ≥ 20g

**Fig. 3:**
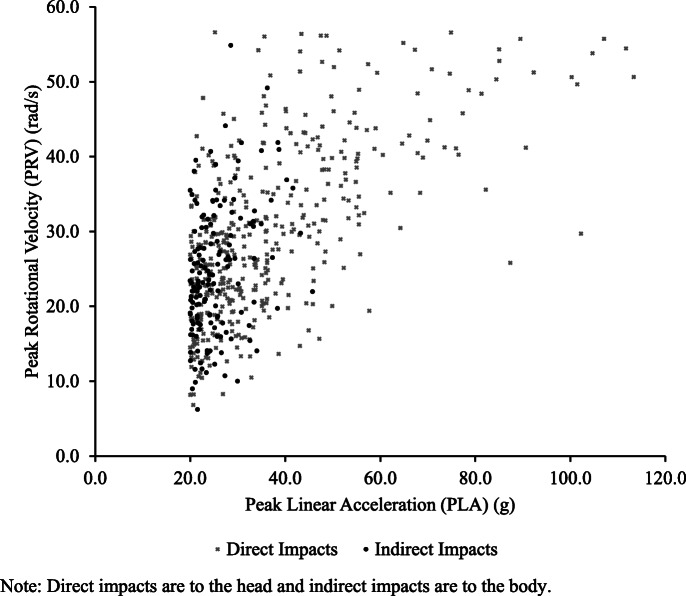
Scatterplot of video verified direct and indirect impacts ≥ 20g recorded by the X-patch™. The multiplication sign denotes Direct Impact. The black circle denotes Indirect Impact

Of the 624 HI ≥ 20g during gameplay, 564 (90.4%) were verified on video. The distribution of all X-patch^TM^-triggered events and VV-HI ≥ 20g can be found in Fig. 2. From 564 VV-HI ≥ 20g, 257 were as a result of a hit-up, 278 from a tackle, and 29 off-the-ball incidents. 413 (73.2%) were identified as direct head impacts and 151 (26.9%) as non-direct impacts occurring to either the shoulder, chest, arm, or waist. Of the 413 direct VV-HI ≥ 20g, the tackler (defender) recorded 204 (49.4%), the ball carrier (attacker) recorded 186 (45.0%), while 23 were recorded during off-the-ball incidents (5.6%; incidental contact *n* = 4; melee/scuffle or fighting *n* = 1; contact celebrating tries *n* = 10; contact celebrating penalty *n* = 1; contact packing scrums *n* = 5; clutching at own head after tackle *n* = 2). Direct VV-HI ≥ 20g (as determined by video review) had a greater PLA compared to indirect VV-HI ≥ 20g [direct *n* = 413, mean = 37.3, median = 31.3, SD = 17.5, range 20–113.3; indirect *n* = 151, mean = 25.5, median = 24.0, SD = 5.4, range 20–45.7; U = 15,728.00, *p* < .001; Cohen’s *d* = 0.83] as well as greater PRV compared to indirect VV-HI ≥ 20g [direct *n* = 413, mean = 29.9, median = 28.5, SD = 11.3, range 6.8-56.6; indirect *n* = 151, mean= 24.8, median = 23.4, SD = 8.7, range 6.2–54.9; *U* = 23,162.00, *p* < .001; *d* = 0.48]. Figure 3 provides a comparison of the peak linear acceleration and peak rotational velocity between all direct and indirect VV-HI ≥ 20g. The individual player data by position, playing time, video-verified impacts, and VV-HI ≥ 20g per game hour are provided in Table 1.

**Table 1:** Cross-tabulation of frequency of verified in-game impacts ≥ 20g measured by the X-patch^TM^

	Playing position	Player time in game (min)	All X-patch^TM^-recorded impacts during game time				X-patch^TM^-recorded direct impacts	
			Sensor-recorded in-game impacts	Video verified game impacts	Percentage of impacts verified (%)	Video verified impacts per game hour	Video verified direct impacts	Video verified direct impacts per game hour
Player 1	Back	211	17	16	94.1	4.5	12	3.4
Player 2	Back	337	22	22	100	3.9	17	3.0
Player 3	Back	490	46	44	95.7	5.4	30	3.7
Player 4	Back	463	36	36	100	4.7	24	3.1
Player 5	Back	300	14	14	100	2.8	9	1.8
Player 6	Back	390	15	15	100	2.3	10	1.5
	Forward	30	2	2	100	4	1	2
Player 7	Forward	259	26	25	96.2	5.8	14	3.2
Player 8	Forward	192	39	35	89.7	10.9	22	6.9
Player 9	Forward	204	67	56	83.6	16.5	44	12.9
Player 10	Forward	398	37	37	100	5.6	31	4.7
Player 11	Forward	272	25	25	100	5.5	20	4.4
Player 12	Forward	247	63	63	100	15.3	45	10.9
Player 13	Back	87	12	11	91.7	7.6	8	5.5
	Forward	63	10	8	80	7.6	7	6.7
Player 14	Forward	118	24	16	66.7	8.1	13	6.6
Player 15	Forward	91	30	26	86.7	17.1	20	13.2
Player 16	Forward	185	62	47	75.8	15.2	37	12
Player 17	Back	180	20	18	90	6	12	4
Player 18	Forward	36	9	8	88.9	13.3	3	5
Player 19	Forward	72	12	11	91.7	9.2	9	7.5
Player 20	Forward	56	26	21	80.8	22.5	17	18.2
Player 21	Back	21	1	0	0	0	0	0
	Forward	60	9	8	88.9	8	7	7
Total		4762	624	564	90.4	7.1	413	5.2

Note: Season totals. Players 6, 13, and 21 played in both forward and back positions during the season. Sensor-recorded impacts were ≥ 20gs

### Impacts Seen on Video and Not Recorded on the Sensors

**Fig. 4:**
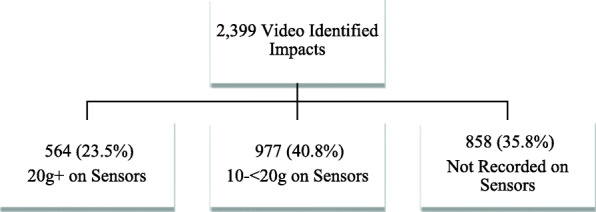
Flow diagram showing video verified impacts

There were 858 video observed impacts, including 28 direct head impacts, that did not result in any triggered event from the X-patch^TM^, either because the sensors did not activate (despite other impacts being recorded on those sensors in close temporal proximity) or because the impact did not reach the 10g threshold (see Fig. 4).

### Sensor-Recorded Impacts Not Verified on Video

**Fig 5:**
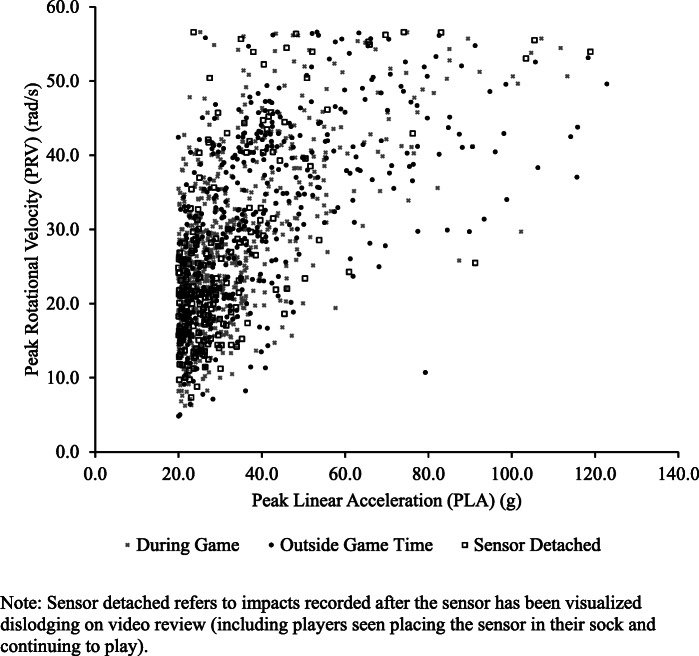
Scatterplot of all impacts ≥ 20g recorded by the X-patch^TM^. The multiplication sign denotes During Game. The black circle denotes Outside Game. The white box denotes Sensor Detached

There were 1199 triggered events that occurred before or after the game which were removed from the analysed data (Fig. 1). There were 506 triggered events registered as ≥ 20g that were not seen on video, and of those 185 occurred before the game (36.6%, presumably during warm up), 60 occurred during the game (11.9%), and 261 occurred after the game (51.6%). Individual impacts during the game, outside game time, and while the sensor was detached are illustrated in Fig. 5.

A total of 60 HI ≥ 20g that occurred during game time were not verified on video review. Of these, 33 HI ≥ 20g were recorded when the player was on the bench, 15 were not visualised (including 12 impacts while the player was not involved or was “behind” the play and three impacts when the game was halted after the awarding of a penalty), one was partially visualised on video but was indeterminant, and 11 recorded impacts had complete visualisation but with no identified contact (including seven during a sharp change of direction from the player, three during change of speed while running, and one with no visible correlate). In all of these instances, there was clearly no contact from another player. Each of these HI ≥ 20g was verified by two reviewers to confirm them as “false-positive” impacts. There was no significant difference in the PLA of VV-HI ≥ 20g versus false-positive HI ≥ 20g [verified *n* = 564, mean=34.1g, median = 28.4g, SD = 16.1, range = 20.0–113.3g; non-verified *n* = 60, mean = 30.9g, median = 26.1g, SD = 13.3, range = 20.0–76.6g; *U* = 14,706.00, *p* = .10; *d* = 0.20] but a difference in PRV [verified mean = 28.5rad/s, median = 26.9rad/s, SD = 10.9, range = 6.2–56.6rad/s; non-verified mean = 25.2rad/s, median = 22.4rad/s, SD = 13.0, range = 6.3–55.4rad/s; *U* = 13,727.00, *p* = .02, *d* = 0.30].

### Situational Characteristics of Video-Verified and Sensor-Recorded Impacts

Of the 413 direct VV-HI ≥ 20g, players sustained an average of 5.2 direct VV-HI ≥ 20g per hour of gameplay, with a slightly higher rate of direct VV-HI ≥ 20g during the second half of the game [first half 4.6 impacts/h, *n* = 192; second half 5.8 impacts/h, *n* = 221]. The magnitude of these direct VV-HI ≥ 20g did not statistically differ between the first and second half (PLA: *U* = 20,211.00, *p* = .41; PRV: *U* = 21,088.00, *p* = .92). Forwards had a higher rate of direct VV-HI ≥ 20g than backs [forwards *M* = 8.08 impacts/h, SD = 4.46; backs *M* = 2.90 impacts/h, SD = 1.60; *t*(22) = 7.58 *p* = .001, *d* = 1.29]. However, the intensity of direct VV-HI ≥ 20g did not statistically differ between forwards and backs (PLA: *U* = 17,047.00, *p* = .53; PRV: *U* = 17,656.00, *p* = .93).

The most common event that caused a VV-HI ≥ 20g was from a defender’s arm directly impacting the head of a ball carrier (*n* = 120). This type of gameplay accounted for 21.3% of all VV-HI ≥ 20g and 46.7% of all hit-up VV-HI ≥ 20g. The most common event associated with a VV-HI ≥ 20g for a tackler was an attacker’s arm (*n* = 60) or waist (*n* = 60) directly impacting the head of the tackler. Each of these accounted for 10.6% of all VV-HI ≥ 20g and 21.6% of tackler VV-HI ≥ 20g. Contact with the playing surface accounted for 44 VV-HI ≥ 20g (7.8%; hit-up *n* = 32, tackle *n* = 9, off-the-ball *n* = 3). Of the 151 indirect VV-HI ≥ 20g, a ball carrier’s shoulder impacting with a defender’s shoulder (11.9%, *n* = 18, 3.2% of all impacts) or the defender’s chest impacting a ball carrier’s shoulder (10.6%, *n* = 16, 2.9% of all impacts) were the most common. A detailed overview of all VV-HI ≥ 20g for a hit-up, tackle, and off-the-ball events is provided in Table 2.

**Table 2:** Sensor-recorded and video-verified impact locations ≥ 20 g

		Impact from opposition (teammate)							
		Head	Shoulder	Chest	Arm	Waist	Leg/knee	Ground	Total
Point of impact	Head	13^a^ (4,7,2)	64^b^ (26,37,1)	34^c^ (12,22,0)	199^d^ (120,60,19)	66^e^ (5,60,1)	26^f^ (13,13,0)	11 (6,5,0)	413 (186,204,23)
	Shoulder	0 (0,0,0)	30 (11,18,1)	29 (16,13,0)	2 (2,0,0)	13 (1,12,0)	0 (0,0,0)	10 (8,1,1)	84 (38,44,2)
	Chest	0 (0,0,0)	26 (10,16,0)	10 (2,8,0)	5^g^ (0,3,2)	0 (0,0,0)	0 (0,0,0)	18 (14,2,2)	59 (26,29,4)
	Arm	0 (0,0,0)	0 (0,0,0)	0 (0,0,0)	0 (0,0,0)	0 (0,0,0)	0 (0,0,0)	1 (1,0,0)	1 (1,0,0)
	Waist	0 (0,0,0)	3 (3,0,0)	0 (0,0,0)	0 (0,0,0)	0 (0,0,0)	0 (0,0,0)	4 (3,1,0)	7 (6,1,0)
	Total	13 (4,7,2)	123 (50,71,2)	73 (30,43,0)	206 (122,63,21)	79 (6,72,1)	26 (13,13,0)	44 (32,9,3)	564 (257,278,29)

Note: Data in the parentheses are for a hit-up, tackle and off-the-ball incident, as follows: (hit-up, tackle, off-the-ball)

^a^ 4 impacts from teammate’s head (3 tackle, 1 off-the-ball)

^b^ 7 impacts from teammate’s shoulder (tackle), 1 impact from player’s own shoulder (hit-up)

^c^ 6 impacts from teammate’s chest (6 tackle)

^d^ 32 impacts from teammate’s arm (20 tackle, 12 off-the-ball), 2 impacts from player’s own arm (2 off-the-ball)

^e^ 5 impacts from teammate’s waist (4 tackle, 1 off-the-ball)

^f^ 2 impacts from teammate’s leg/knee (2 tackle)

^g^ 1 impacts from teammate’s arm (off-the-ball)

### Direction of Sensor-Recorded and Direct VV-HI ≥ 20 g

When looking at the location of all direct VV-HI ≥ 20g (*n* = 413) via the IMS, most occurred to the front (*n* = 198; 47.9%), followed by the side (*n* = 111; 26.9%), back (*n* = 83; 20.1%), and top (*n* = 21; 5.1%) of the head. When examining the location via video review, we found most direct VV-HI ≥ 20g occurred to the side (*n* = 357, 86.4%) with fewer to the front (*n* = 14, 3.4%), back (*n* = 34, 8.2%), and top (*n* = 8, 1.9%) of the head. The location of direct VV-HI ≥ 20g corresponded in only 24.9% of cases with the video review [42.9% (*n* = 6) to the front of the head, 25.2% (*n* = 90) to the side, 17.6% (*n* = 6) to the back, and 12.5% (*n* = 1) to the top]. The sensor-derived impact location was poorly correlated with side-on direct head impacts visualised on video, and as such overestimated VV-HI ≥ 20g in all other directions, particularly front-on impacts. A detailed description of the location accuracy for direct and indirect VV-HI ≥ 20g is provided in Table 3.

**Table 3:** Video verified impacts: location accuracy of direct and indirect impacts ≥ 20g

	Total				Direct impact				Indirect impact			
	X-patch^TM^ (*n*)	Video (*n*)	Agreement (*n*)	Accuracy (%)	X-patch^TM^ (*n*)	Video (*n*)	Agreement (*n*)	Accuracy (%)	X-patch^TM^ (*n*)	Video (*n*)	Agreement (*n*)	Accuracy (%)
Front	269	68	35	51.5	198	14	6	42.9	71	54	29	53.7
Side	150	447	118	26.4	111	357	90	25.2	39	90	28	31.1
Back	116	41	9	22.0	83	34	6	17.6	33	7	3	42.9
Top	29	8	1	12.5	21	8	1	12.5	8	0	0	0
Total	564	564	163	28.9	413	413	103	24.9	151	151	60	39.7

### Tackles and Secondary Impacts

Secondary impacts during a tackle (i.e. impacts after the initial contact in the same tackle event) accounted for 46.1% (*n* = 260) of total VV-HI ≥ 20g and 53.5% (*n* = 221) of all direct VV-HI ≥ 20g. For 260 secondary impacts, 16.2% (*n* = 42) were accompanied by a VV-HI ≥ 20g. There were 456 tackles that resulted in the 535 VV-HI ≥ 20g, excluding impacts that occurred “off the ball.” For 388 tackles there was one impact recorded, for 60 there were two impacts recorded, for 7 there were three impacts recorded, and for 1 there were six impacts recorded. There were an additional 27 incidents occurring off the ball that resulted in 29 VV-HI ≥ 20g (25 with one impact recorded and 2 with two impacts). The hit-up was a play that accounted for approximately 47.1% (*n* = 215) of all X-patch^TM^-recorded events. Of the hit-up plays, the forward positions accounted for 62.8% (*n* = 135) of those impacts, while the back positions accounted for approximately 37.2% (*n* = 80) of impacts. The tackle accounted for approximately 52.9% (*n* = 241) of all X-patch^TM^-recorded events. Of the tackles, the forward positions accounted for 73% (*n* = 175) of those impacts, while the back positions accounted for approximately 27% (*n* = 66) of impacts.

## Discussion

This study is the first to analyse video and verify X-patch^TM^-recorded data in elite youth rugby league players. VV-HI ≥ 20g accounted for 90.4% of all HI ≥ 20g during game time, revealing a high rate of agreement, similar to rates previously recorded in men’s semi-professional rugby league [17]. Cross-verification with a secondary source, such as video review, is highly recommended to reduce false-positive readings that may inflate players’ cumulative and average PLAs across a season. Of particular importance is the risk for fundamentally misinterpreting the highest acceleration readings, because those could be falsely attributed to high energy head impacts. Of the 45 impacts recorded as 80gs or greater, only 35.6% occurred during the game, while 55.6% were outside game time, and 8.9% were recorded while the sensor was known to be detached (Fig. 5). When viewing video footage during one of our prior studies, we saw one example when six 40g or higher “head impacts” (ranging from 40.6 to 58.8g) were recorded after a game when two players were shaking hands and one player tapped the side of the other players head, during the handshake, presumably directly on the sensor [17].

Upon video review, we discovered not all recorded “head impacts” occurred as a result of a direct impact to the head. We recorded 5.2 direct VV-HI ≥ 20g per game hour which is similar to that recorded previously in men’s semi-professional rugby (6.0 direct VV-HI ≥ 20 g/h) [17]. Although the majority of VV-HI ≥ 20g occurred as a result of a direct force to the head (73.2%), the X-patch^TM^-recorded impacts confirmed by video to be caused by an impulsive force to the head after an impact elsewhere on the body (i.e. chest/torso, shoulder, arm, etc.). The rate of direct VV-HI ≥ 20g was not significantly different between the first and second half, but showed a greater exposure experienced by forwards when compared to backs, consistent with previous literature [17, 32, 33] (Table 4). There was poor agreement in the location and direction of VV-HI ≥ 20g between the X-patch^TM^ and video review. This is consistent with in vivo laboratory findings which found skin-mounted sensors showed large measurement errors, with acceleration peaks in different directions from the same impacts recorded with mouthguard-based sensors [26]. For direct VV-HI ≥ 20g, the X-patch^TM^ accurately recorded the location in 24.9% (*n* = 103) of impacts, see Table 3. Previously, Kuo and colleagues, using a similar tri-axial linear accelerometer embedded into a mouthguard, reported similarly poor rates of agreement between sensor-recorded and video-identified impacts (37.3%), with impact locations that did not match the visualised head kinematics [34].

**Table 4:** Frequency of video verified direct head impacts by game time and playing position

		Sensor recorded and video verified ≥ 20 g		Verified direct head impacts ≥ 20 g	
	Playing hours (min)	Game impacts (*n*)	Impacts per game hour (*n*)	Game impacts (*n*)	Impacts per game hour (*n*)
1st half	41.3 (2479)	271	6.6	192	4.6
2nd half	38.1 (2283)	293	7.7	221	5.8
Forwards	38.1 (2283)	388	10.2	291	7.6
Backs	41.3 (2479)	176	4.3	122	3.0
Total sample	79.4 (4762)	564	7.1	413	5.2

Note: The total number of impacts by playing positions (forwards/backs) was divided by the total minutes played by each playing position

It is important that when sensor data are collected, they are closely analysed because there is potential for gross over-estimation of head impacts if not carefully processed to remove triggered events outside of gameplay (Fig. 5) [17, 18]. Of all triggered events ≥ 20g (*n* = 1,291), only approximately half (*n* = 624, 48.3%) occurred during the game, with the majority of impacts occurring either before or after the game or after a sensor had become detached from the head (Fig. 4). Upon video review, there were multiple triggered events (*n* = 22) after the sensor was seen detaching from a player, presumably from the sensor hitting the ground after falling or players stepping on it during the game. On two occasions, after the X-patch^TM^ became dislodged, the players can be clearly visualised placing the sensor in their sock until the conclusion of the game, leading to a large number of triggered events ≥ 20g (*n* = 199) being recorded from running.

In this study, we employed a video verification approach with two reviewers independently reviewing 60 HI ≥ 20g that occurred during game time that were not verified by video. This was used for quality assurance to verify false-positive readings so that they could be confidently removed from head impact exposure data prior to analysis. Interestingly, upon close review of non-verified impacts, we discovered 33 impacts that occurred while the player was on the sideline. It is unclear how or why these impacts were recorded on the sensors because there was no available video of players on the sidelines. It is possible that these impacts occurred while players were preparing to enter the game by simulating tackles. Previously Cortes and colleagues [18], in a review of impacts in collegiate lacrosse, reported that 99 impacts ≥ 20g occurred on the sideline. This suggests that a secondary source, such as video review, is important when trying to quantify in-game recorded head impacts to ensure reported impacts occurred while the player was involved in the game. If the non-game data is not removed, it would artificially inflate the number of head impacts a player sustains. There were also a number of clear head impacts seen on video review that did not register on the sensor, consistent with previous X-patch^TM^ studies [20, 35]. There were a total of 2399 video-identified impacts to the head or body. Of these, the authors of this study visualised 858 (35.8%) impact events on video review that were either not recorded at all by the sensor, or that registered as less than 10g, of which there were many observable direct head impacts (Fig. 2). The exact number of impacts is difficult to determine and may be higher because this number captures the number of events (e.g. tackles), and multiple impacts may have occurred in each event. We also found a large number of impacts were secondary impacts during tackle events that occurred after the initial contact with 46.3% of all verified impacts coming after the initial impact was seen on video. Of these secondary impacts, 83.8% occurred after the primary impact was either not recorded by the sensor or registered under the 20g threshold. Having multiple impacts in tackle events makes it difficult to determine exactly how many “larger” impacts identifiable on video review were not registered by the X-patch^TM^.

### Limitations

This study consisted of a relatively small sample size, and no player played all games. Due to faulty equipment at the end of the season, the number of working sensors was less than the number of participating players in each game. Because of this, sensors were given to players likely to play more minutes throughout the game to maximise data collection. Further, due to time and personnel constraints, training sessions and two games throughout the season were unable to be staffed by research personnel; these data underrepresent the total accumulation of head impacts over the course of a full season. Additionally, our statistical analyses (i.e. *t* tests and Mann-Whitney *U* tests) were exploratory and should be replicated in larger, more representative samples. Similar to our previous study [17], false-negative incidences only included the initial impact as a single “missed” impact when there likely were more subsequent, or secondary, impacts also not registered by the sensor. Although we double verified each false-positive impact to ensure accuracy, all other impacts were coded by a single researcher creating a possibility of some impacts being missed due to human error.

This study utilised one high-definition sideline video camera that panned across the entire field which limits the ability to accurately verify impacts and signs of concussion. Some impacts may be obstructed from view by another player, and thus, the exact location of some impacts may be inaccurate. Research studies on professional sports with multiple camera angles may be more accurate in analysing video signs of concussion and determining location of verified impacts. Critically, the process for synchronising, and interpreting, the video and X-patch^TM^ data in a sport where continuous impacts are present is challenging. Given the volume of impact data collected from the X-patch^TM^ when no apparent contact was observed (i.e. high sampling rate of the X-patch^TM^ in the absence of verified impact), in combination with a sport that has a high frequency of body contact, it seems likely that the sensor is recording triggered events that are not actually impacts to the head or body [20, 29].

## Conclusion

The findings from this study are consistent with previous research, highlighting the importance of using video review as the primary source of information on head impact and supplementing that with X-patch^TM^-recorded data. That is, the X-patch^TM^ has serious limitations as a primary data source [17, 18, 20, 27]. The current study identified similar high rates of false-positive direct head impacts recorded by the wearable sensors in junior representative rugby league as were previously described in semi-professional men’s rugby league, Australian Football, and collegiate lacrosse respectively. Although there is potential for impact sensors to play an assistive role to medical staff, more research is needed to ensure the accuracy of data collected and to establish their usefulness for injury surveillance. With ongoing improvements in methodology and technology, future impact sensors might provide important information for junior or amateur sports in recording player exposure rates and differentiating between incidental movements and actual collisions.

## Data Availability

The data generated during the current study are not publicly available due to the institutional human ethics committee approval requirements that “only the researchers listed as investigators on the ethics application will have access to the data” but may be available from the corresponding author on reasonable request.

## Abbreviations

HI ≥ 20g: Head impact ≥ 20g
HIA: Head injury assessment
HIE: Head impact events
IMS: Injury management software
NRL: National Rugby League
NSW: New South Wales
PLA: Peak linear acceleration
PRA: Peak rotational acceleration
PRV: Peak rotational velocity
SD: Standard deviation
TM: Trademark
VV-HI ≥ 20g: Video-verified head impact ≥ 20g
